# Comparison of Adverse Reactions Between Remimazolam and Propofol in Hysteroscopic Surgery in Mainland China: A Meta‐Analysis and Systematic Review

**DOI:** 10.1155/anrp/4206976

**Published:** 2026-01-06

**Authors:** Zirui Liu, Kexu Zhu, Zidong Zhao, Qi Zeng

**Affiliations:** ^1^ Xiangya School of Medicine, Central South University, 172 Tongzipo Road Yuelu District, Changsha, 410013, Hunan, China, csu.edu.cn

**Keywords:** anesthetic, hysteroscope, meta-analysis, propofol, remimazolam, surgery

## Abstract

**Background:**

The purpose of this meta‐analysis was to compare the adverse reactions of remimazolam and propofol in hysteroscopic anesthesia and to evaluate the efficacy of remimazolam in alleviating adverse reactions.

**Methods:**

We conducted a database search using China National Knowledge Infrastructure (CNKI), PubMed, and the Cochrane Library to identify randomized controlled trials (RCTs) comparing the use of remimazolam with propofol for hysteroscopy procedures. We extracted data on the occurrence of adverse reactions such as hypotension, respiratory depression, dizziness, nausea, and vomiting for meta‐analysis. Literature published until July 2024 was screened from each database, and the quality of the included studies was assessed using the bias risk assessment tool recommended by Cochrane. Data analysis was performed using RevMan 5.3 software, developed by the Cochrane Collaboration in the United Kingdom.

**Results:**

The pooled results demonstrated significant differences in favor of remimazolam when compared to propofol for procedural sedation. Patients receiving remimazolam showed a lower risk of respiratory depression (RR: 0.19; 95% CI: [0.11, 0.33]; *I* ^2^ = 0%; *p* < 0.00001), dizziness (RR: 0.10; 95% CI: [0.04, 0.31]; *I* ^2^ = 0%; *p* < 0.0001), and postoperative nausea and vomiting (RR: 0.60; 95% CI: [0.15, 2.46]; *I* ^2^ = 0%; *p* = 0.48). No significant differences were observed in anesthesia recovery time between the remimazolam and propofol groups (MD: −0.07; 95% CI: [−0.18–1.04]; *I* ^2^ = 98%; *p* = 0.90).

**Conclusion:**

Compared to propofol, remimazolam reduces the occurrence of hypotension, respiratory depression, dizziness, and postoperative nausea and vomiting in the anesthesia management of hysteroscopy procedures.

## 1. Introduction

Hysteroscopy is among the most common minimally invasive diagnostic and therapeutic techniques for uterine diseases [1, 2]. With the advancement of assisted reproductive technology and minimally invasive techniques, as well as increased patient demands for comfortable medical procedures, outpatient painless hysteroscopy is becoming increasingly common [3, 4].

Propofol, combined with opioids, is commonly employed for sedation during hysteroscopic examinations [5, 6]. Propofol offers a rapid onset, convenient titration, and short half‐life [7]. However, propofol use is associated with pain on injection, postoperative dizziness, and respiratory depression [8, 9].

Remimazolam is a novel intravenous benzodiazepine. Like propofol, remimazolam has a rapid onset and short duration of action. Owing to its ester metabolism (similar to remifentanil), remimazolam does not accumulate in tissues and is metabolized independently of liver and kidney function. Remimazolam is widely used for both sedation as well as anesthesia induction and maintenance [10]. On July 2, 2020, the U.S. FDA approved the market release of remimazolam for sedation during medical procedures (lasting up to 30 min) in adult patients [11, 12].

Remimazolam was approved for use in China in 2019, where it is manufactured by two pharmaceutical companies. A substantial amount of clinical data and usage experience has been published since its introduction. Given that China is a multiethnic country, the extensive patient data obtained from its use holds certain representativeness, particularly for Japan and South Korea, whose populations predominantly identify as Asian.

This meta‐analysis compares the clinical and adverse effects of remimazolam and propofol in patients requiring sedation for hysteroscopy.

## 2. Research Methods and Materials

We conducted a systematic review and meta‐analysis based on the Cochrane Intervention Measures Handbook. We presented our findings using the preferred reporting items outlined in the Preferred Reporting Items for Systematic Reviews and Meta‐Analyses (PRISMA) guidelines. We registered this study with PROSPERO with the registration ID: CRD42023464718.

### 2.1. Search Strategy and Selection Criteria

To retrieve relevant literature databases online, we utilized several English resources, including PubMed and the Cochrane Library, as well as the Chinese database China National Knowledge Infrastructure (CNKI). Our search was conducted from the date of product approval through July 2024. In the English databases, we used the search query “(Remimazolam) AND (hysteroscopy)” for retrieval purposes, while in the Chinese database, we applied the search query “((Remimazolam) AND (propofol)) AND (hysteroscopy).”

### 2.2. Eligibility Criteria

The six predefined criteria for evidence selection were as follows: (1) randomized controlled trials (RCTs), (2) studies that involved adult patients older than 18 years who underwent medical procedures with procedural sedation using propofol or remimazolam, (3) studies that reported at least one comparative outcome of remimazolam and propofol, (4) the patient underwent hysteroscopy, (5) the sample size was at least 20, (6) article written in English or Chinese, and (7) patients from mainland China.

### 2.3. Exclusion Criteria

The studies were considered ineligible when any of the following criteria were met:

(1) They were review articles, case reports, case series, retrospective data analysis, or nonrandomized prospective studies.
(2) The data in the articles could not be pooled or were not relevant, thus rendering the study unsuitable for meta‐analysis.
(3) The trials compared propofol with other sedatives.
(4) Duplicate searches across different databases.

### 2.4. Data Extraction and Quality Evaluation

We extracted the data from eligible articles, including (1) the first author’s name, (2) publication year, (3) country, (4) American Society of Anesthesiology (ASA) score, (5) sample size, (6) procedure type, and (7) protocol of procedural sedation.

### 2.5. Assessment of Risk of Bias

To classify each article as low or high risk of bias or unclear risk of bias, we used the Cochrane Collaboration tool, which involved 7 items to evaluate the risk of bias.

### 2.6. Statistical Analysis

We used Review Manager V5.3 to perform the statistical analysis. Categorical variables were expressed as the relative risk (RR) with a 95% confidence interval (CI), while continuous variables were expressed as the standardized mean difference (SMD) with a 95% CI.

To address potential sensitivity and heterogeneity concerns, a model‐altering approach was implemented in this study. The *I* ^2^ statistic was utilized to assess heterogeneity, whereby an *I* ^2^ value exceeding 50% indicated high heterogeneity and led to the adoption of a random‐effects model. Conversely, if the *I* ^2^ value fell below 50%, it indicated lower heterogeneity, prompting the use of a fixed‐effects model. Publication bias was assessed using the Cochrane Collaboration risk of bias tool. Funnel plots were used to assess the goodness of fit.

### 2.7. Outcomes

Our primary objective was to compare the safety of remimazolam and propofol by analyzing the incidence of adverse events associated with their use. These adverse effects included hypotension, respiratory depression, dizziness, and postoperative nausea and vomiting. In addition, we evaluated the time to recovery from anesthesia as a secondary outcome to show the perioperative effects of the two anesthetics.

## 3. Results

### 3.1. Study Selection

**Figure 1:**
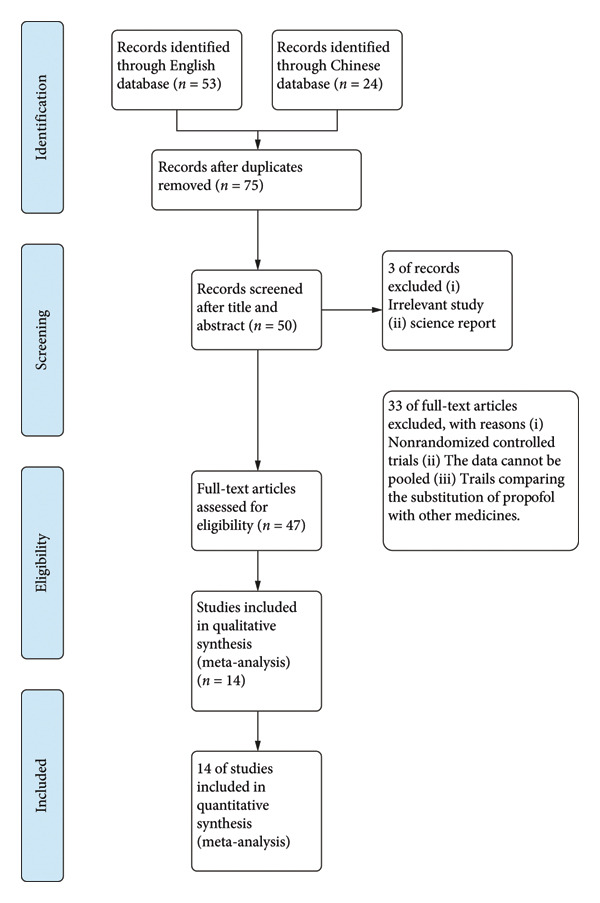
PRISMA flowchart diagram. We initially extracted 75 potential references. Screening the titles and abstracts yielded 47 full‐text articles, the eligibility of which was assessed. Eventually, 14 studies were included for qualitative and quantitative syntheses. PRISMA = Preferred reporting items for systematic reviews and meta‐analyses.

Figure 1 illustrates the detailed flowchart of article selection. We retrieved 53 articles from the English database. We retrieved 24 articles from the Chinese database. Two of the Chinese articles were excluded due to duplicate searches. We excluded 25 articles describing animal experiments. We excluded 33 of the remaining papers as they did not meet the inclusion criteria for analysis.

### 3.2. Study Characteristics

In the end, we included 14 RCTs involving 633 subjects in the meta‐analysis, and the details are demonstrated in Table 1 [13–27]. The publication year of the included studies ranged from 2021 to 2024. All enrolled patients met the screening criteria.

**Table 1:** Characteristics of the included studies in the meta‐analysis.

Study	Patients (T vs. C)	ASA	Intervention	Adverse reaction
Li [17]	50 vs. 50	I‐II	Propofol 4.0 mg/kg 40.0 mg/min	Respiratory depression; hypotension; dizziness; PONV
			Remi^t^ 0.1 mg/kg 40.0 mg/min	

Li et al. [20]	50 vs. 50	I‐II	Propofol 1.5 mg/kg 2.0–5.0 mg/kg/h	Respiratory depression; dizziness
			Remi^b^ 6.0 mg/kg/h	

Zhang et al. [25]	64 vs. 64	I‐II	Propofol 1.5 mg/kg 4.0–10.0 mg/kg/h	Respiratory depression; hypotension; dizziness; PONV
			Remi^t^ 0.2 mg/kg 0.4–1.0 mg/kg/h	

Zhang et al. [27]	41 vs. 41	I‐II	Propofol 1.5–2.0 mg/kg 3.0–6.0 mg/kg/h	Respiratory depression; hypotension; dizziness
			Remi^b^ 0.2 mg/kg 1 mg/kg/h	

Zhang et al. [26]	30 vs. 30	I‐II	Propofol 2.0 mg/kg 5.0 mg/kg/h	Respiratory depression; PONV
			Remi^t^ 0.25 mg/kg 0.48 mg/kg/h	

Zheng et al. [23]	40 vs. 40	I‐II	Propofol 2.0 mg/kg 6.0 mg/kg/h	Respiratory depression; PONV
			Remi^t^ 0.2 mg/kg 2.5 mg each time	

Wu et al. [22]	56 vs. 56	I‐II	Propofol 2.0 mg/kg 4.0 mg/kg/h	Respiratory depression; hypotension; dizziness; PONV
			Remi^b^ 0.4 mg/kg 1 mg/kg/h	

Wang et al. [21]	43 vs. 43	I‐II	Propofol 2.0 mg/kg/h	Respiratory depression; dizziness; PONV
			Remi^n^ 0.4 mg/kg 1 mg/kg/h	

Li et al. [20]	41 vs. 41	I‐II	Propofol 2.0 mg/kg/h	Respiratory depression; dizziness; PONV
			Remi^n^ 0.3 mg/kg 1 mg/kg/h	

Qing et al. [19]	40 vs. 40	I‐II	Propofol 2.0 mg/kg 0.4 mg/kg each time	Dizziness
			Remi^b^ 0.2 mg/kg 2.5 mg each time	

Peng [18]	41 vs. 41	I‐II	Propofol 2.0 mg/kg 0.5 mg/kg each time	Respiratory depression; dizziness; PONV
			Remi^n^ 0.2 mg/kg 2.5 mg each time	

Li et al. [15]	63 vs. 63	I‐II	Propofol 0.5–1.5 mg/kg 3.0–5.0 mg/kg/h	Hypotension; PONV
			Remi^b^ 0.05–0.15 mg/kg 0.3–0.5 mg/kg/h	

Jiang et al. [13]	40 vs. 40	I‐II	Propofol 4.0 mg/kg/h	Respiratory depression; hypotension; PONV
			Remi^b^ 1.0 mg/kg/h	

Li et al. [16]	59 vs. 59	I‐II	Propofol 1.5–2.0 mg/kg 0.3–0.5 mg/kg each time	Hypotension; PONV
			Remi^t^ 0.15–0.2 mg/kg 2.5 mg each time	

*Note:* T vs. C = treatment group vs. control group.

Abbreviations: ASA = American Society of Anesthesiologists classification, PONV = postoperative nausea and vomiting, remi^b^ = remimazolam besilate, remi^n^ = substituent not reported, remi^t^ = remimazolam tosilate.

### 3.3. Risk of Bias in the Included Studies

**Figure 2:**
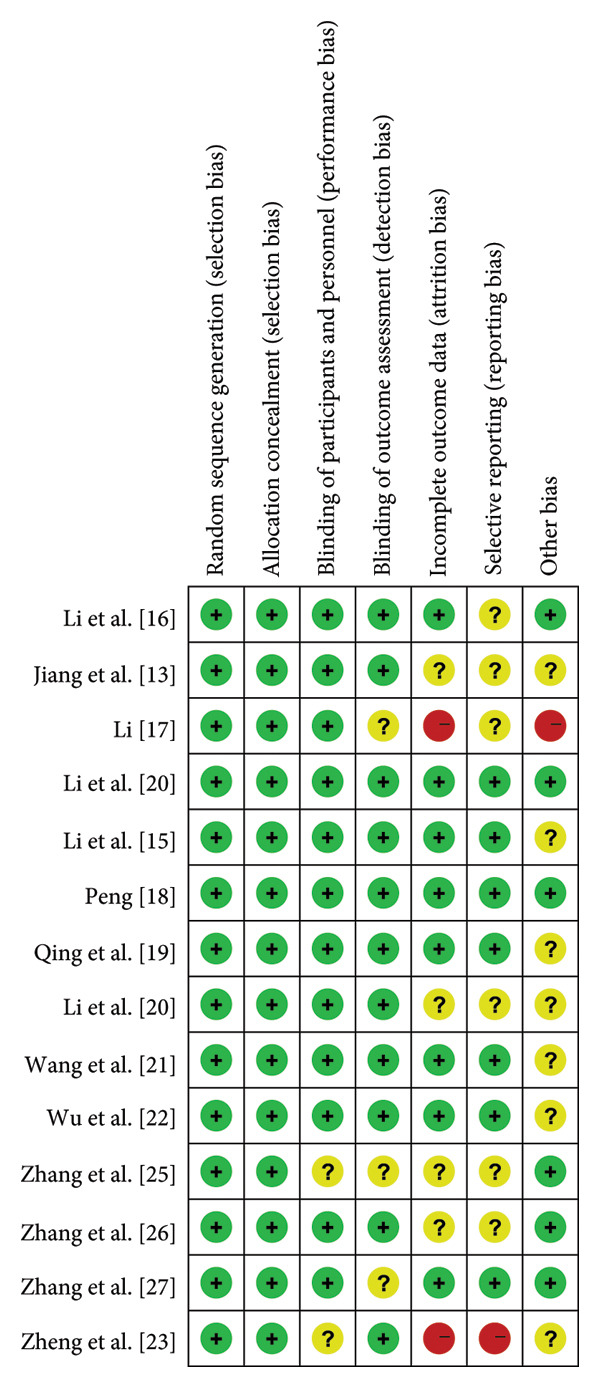
Risk of bias graph: review authors’ judgments about each risk of bias item presented as percentages across all included studies. + = Low risk; ? = unclear; − = High risk.

We categorized seven articles as having an “ambiguous risk of bias” because they did not clearly state participant blinding and the utilization of blind assessment methods for evaluating results (Figure 2). These seven articles included RCTs with comprehensive data on relevant symptoms and originated from significant hospitals across China; we evaluated the potential for incomplete or selective publication of data, as well as the risks associated with withdrawal and reporting biases. For those articles that have a high risk of bias when evaluated using RevMan software, as well as those that do not meet the evaluation criteria of this study in terms of experimental design, they were not included in this meta‐analysis. This evaluation took into account the influence of the hospitals involved and the quality of the journals in which the RCTs were published (refer to Figure 2).

### 3.4. Safety and Efficacy of Remimazolam Versus Propofol

**Figure 4:**
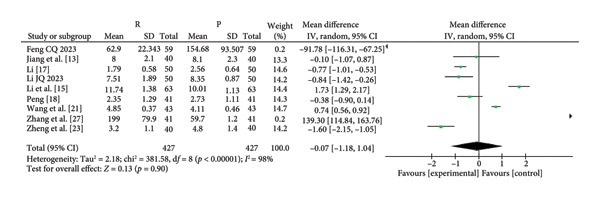
Forest map of remimazolam and propofol anesthesia recovery time.

The pooled results demonstrated that patients given remimazolam for procedural sedation had a lower risk of hypotension (RR: 5.25; 95% CI: [2.63, 10.48]; *I* ^2^ = 0%; *p* < 0.00001), respiratory depression (RR: 0.19 95% CI: [0.11, 0.33]; *I* ^2^ = 0%; *p* < 0.00001), dizziness (RR: 0.10; 95% CI: [0.04, 0.31]; *I* ^2^ = 0%; *p* < 0.0001), and postoperative nausea and vomiting (RR: 0.60; 95% CI: [0.15, 2.46]; *I* ^2^ = 0%; *p* = 0.48) (Figure 3). The characteristics of the included studies in the meta‐analysis are listed in Table 1. Among the five indicators of adverse reactions, only nausea and vomiting had no significant statistical significance (*p* slightly greater than 0.05). No obvious differences were observed in anesthesia recovery time (MD: −0.07; 95% CI: [−0.18–1.04]; *I* ^2^ = 98%; *p* = 0.90; Figure 4) between the remimazolam and propofol groups and the procedure duration. The *I* ^2^ value for the anesthesia recovery time reached 98%, indicating a significant heterogeneity in this measure. The aforementioned adverse reaction results exhibited no considerable heterogeneity, and the included RCTs displayed no significant disparities concerning age, gender, ASA, and other collected data. Moreover, there was no evidence suggesting a significant distinction in clinical outcomes between these two drugs. We tried to exclude some included references to test sensitivity, but the statistical significance did not change after the exclusion of some references, so the sensitivity was considered low.

Figure 3Specific forest map of adverse reactions of remimazolam and propofol. (a) Hypotension. (b) Respiratory depression. (c) Dizziness. (d) Postoperative nausea and vomiting.

**Figure:**
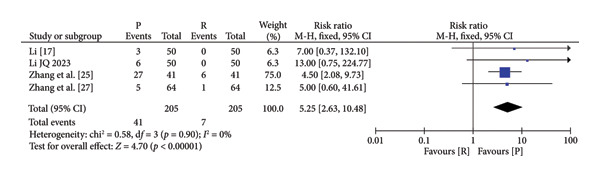
(a)

**Figure:**
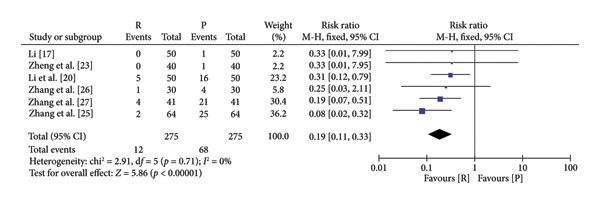
(b)

**Figure:**
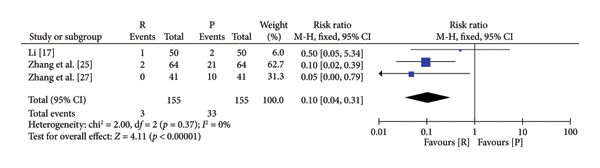
(c)

**Figure:**
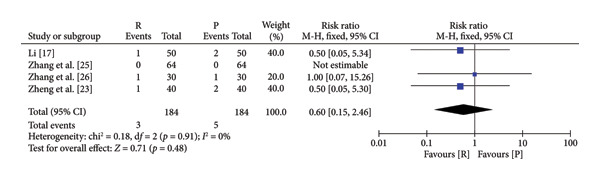
(d)

### 3.5. Publication Bias

To assess publication bias, the Cochrane Collaboration risk of bias tool was used, and the funnel plot was also used to assess publication bias based on the primary outcome. The two funnel plots for the respiratory depression and hypotension indexes in this study were visually asymmetrical around the midline, indicating the presence of publication bias in these studies. The funnel plots of dizziness and postoperative nausea and vomiting were visually symmetrical to the midline, so publication bias was not considered (Figure 5).

Figure 5The results of the funnel plot. (a) Hypotension. (b) Respiratory depression. (c) Dizziness. (d) Postoperative nausea and vomiting.

**Figure:**
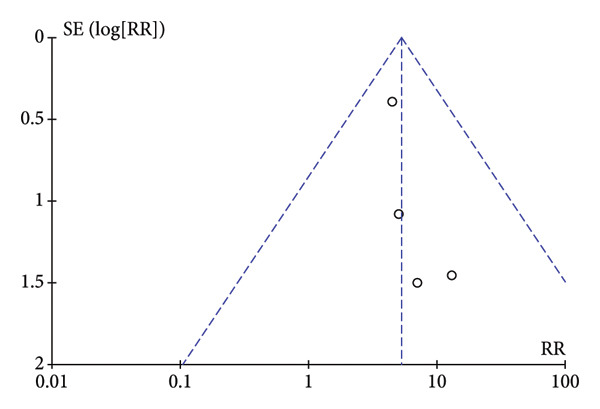
(a)

**Figure:**
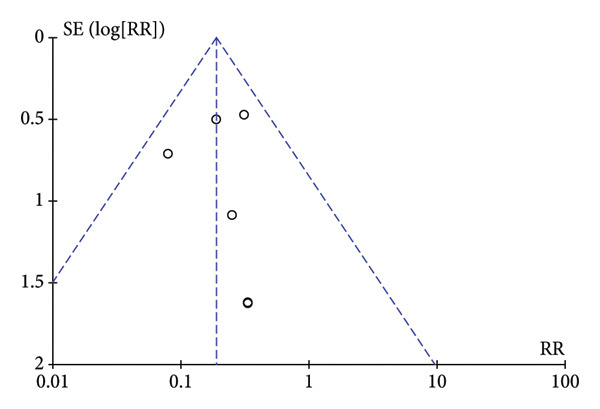
(b)

**Figure:**
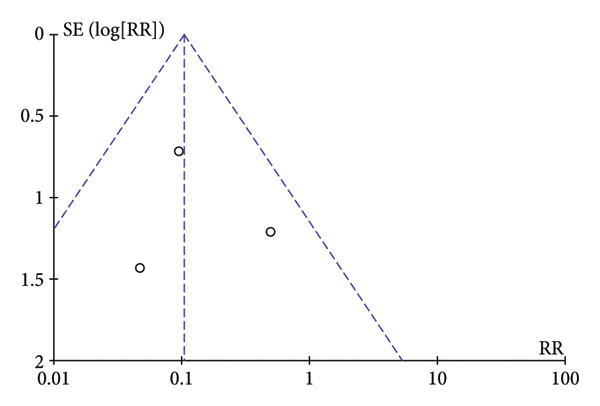
(c)

**Figure:**
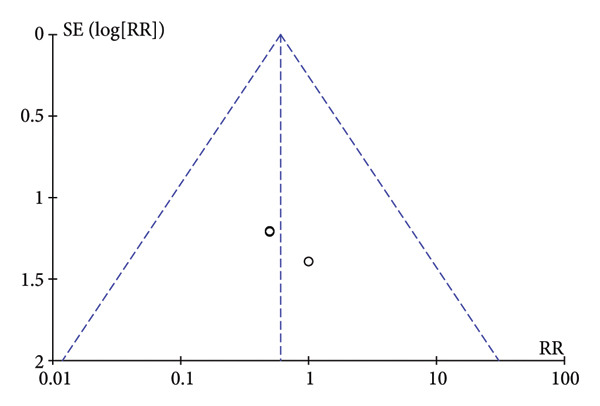
(d)

## 4. Discussion

When utilized for anesthesia during hysteroscopy, remimazolam demonstrates a lower incidence of adverse reactions compared to propofol. It effectively mitigates postanesthetic complications, including hypotension, respiratory depression, dizziness, and postoperative nausea and vomiting, making it a viable choice for hysteroscopy procedures. Consistent with previous literature [28], we found no significant disparity in the awakening time between remimazolam and propofol. These data suggest that remimazolam is a better option for sedation in patients undergoing hysteroscopy, and likely for other endoscopic procedures as well.

Although injection pain during anesthesia is not considered a major complication, it can cause discomfort and anxiety in patients. Women who undergo hysteroscopy, in particular, often express concern about potential pain and discomfort during the procedure. A large body of literature and clinical practices have confirmed that propofol is significantly associated with injection pain, while remimazolam is an ester benzodiazepine containing different components, which can theoretically reduce injection pain [29–32]. Our research findings substantiate the idea that remimazolam significantly reduces injection pain compared to propofol, providing patients with an enhanced experience and aligning with the principles of painless surgery.

The *I* ^2^ value for the anesthesia recovery time reached 98%, indicating a significant heterogeneity in this measure. This could be attributed to the different types of intravenous anesthetics used during surgery, as well as variations in the dosages maintained for each anesthetic during the procedure, resulting in the observed heterogeneity. In addition, individual differences among patients and variations in their ability to metabolize anesthetics may also contribute to the heterogeneity. However, since the patients included in this study did not exhibit significant differences in terms of age and gender, it is believed that the possibility of individual differences causing the observed heterogeneity is relatively low. Moreover, the varying methods of drug combinations used in anesthesia presented challenges in merging identical data.

Remimazolam carries a higher economic burden in comparison to the currently utilized anesthetic drugs for hysteroscopy. This may impose financial constraints on Chinese patients. The clinician should discuss the cost of remimazolam compared to propofol with patients to determine whether the advantages associated with remimazolam justify the additional cost.

The 14 RCTs included in our analysis were conducted across Mainland China. As a multiethnic country in Asia, this study, to some extent, reflects the influence of racial factors on treatment outcomes among Asian patients, thereby holding a certain level of representativeness and practical significance. However, in broader regions across other continents, more specific evaluations are needed based on their unique clinical practices.

## 5. Conclusion and Limitation

Since remifentanil has only been available in China for 6 years, there are only a few RCTs that meet our criteria. Second, our primary focus was to compare adverse reactions associated with remimazolam and propofol.

In conclusion, remimazolam is associated with a similar onset and offset to propofol when used for hysteroscopy sedation; however, it is associated with fewer adverse effects.

## Ethics Statement

No ethical approval was required for the present study since the data were solely collected from CNKI, PubMed, and Cochrane Library databases.

## Conflicts of Interest

The authors declare no conflicts of interest.

## Author Contributions

Zirui Liu: conceptualization, validation, data extraction and curation, methodology and software search, initial drafting, figure generation, article revisions, and project management. Kexu Zhu: initial drafting, writing of comments, and editing. Zidong Zhao: supervision, investigation, and data curation. Qi Zeng: content guidance and English language editing.

## Funding

No funding was received for this study.

## Data Availability

Data from this clinical study can be accessed by requesting the same from the corresponding author through e‐mail.

## References

[1] Okohue J. E. , Overview of Hysteroscopy, West African Journal of Medicine. (2020) 37, no. 2, 178–182. 32150637

[2] Cela V. , Litta P. , Franchini M. et al., Fertility-Enhancing Hysteroscopic Surgery, Minerva Ginecologica. (2016) 68, no. 2, 167–174. 26928416

[3] Bettocchi S. , Achilarre M. T. , Ceci O. , and Luigi S. , Fertility-Enhancing Hysteroscopic Surgery, Seminars in Reproductive Medicine. (2011) 29, no. 2, 75–82, 10.1055/s-0031-1272469, 2-s2.0-79953197597. 21437821

[4] Murdoch J. A. and Gan T. J. , Anesthesia for Hysteroscopy, Anesthesiology Clinics of North America. (2001) 19, no. 1, 125–140, 10.1016/s0889-8537(05)70215-7, 2-s2.0-0035123772. 11244913

[5] Knigge S. and Hahnenkamp K. , Nonoperating Room Anesthesia for Endoscopic Procedures, Current Opinion in Anaesthesiology. (2017) 30, no. 6, 652–657, 10.1097/aco.0000000000000518, 2-s2.0-85033779785. 28938299

[6] Cho Y. J. , Role of Anesthesia in Endoscopic Operations, Gastrointestinal Endoscopy Clinics of North America. (2021) 31, no. 4, 759–772, 10.1016/j.giec.2021.05.011. 34538414

[7] Ferrier D. C. , Kiely J. , and Luxton R. , Propofol Detection for Monitoring of Intravenous Anaesthesia: A Review, Journal of Clinical Monitoring and Computing. (2022) 36, no. 2, 315–323, 10.1007/s10877-021-00738-5. 34213720 PMC9123036

[8] Nishizawa T. and Suzuki H. , Propofol for Gastrointestinal Endoscopy, United European Gastroenterology Journal. (2018) 6, no. 6, 801–805, 10.1177/2050640618767594, 2-s2.0-85044570574. 30023057 PMC6047291

[9] Stogiannou D. , Protopapas A. , Protopapas A. , and Tziomalos K. , Is Propofol the Optimal Sedative in Gastrointestinal Endoscopy?, Acta Gastro-Enterologica Belgica. (2018) 81, no. 4, 520–524. 30645922

[10] Kilpatrick G. J. , Remimazolam: Non-Clinical and Clinical Profile of a New Sedative/Anesthetic Agent, Frontiers in Pharmacology. (2021) 12, 10.3389/fphar.2021.690875. PMC8329483 34354587

[11] Keam S. J. , Remimazolam: First Approval, Drugs. (2020) 80, no. 6, 625–633, 10.1007/s40265-020-01299-8. 32274703

[12] Wang M. , Zhao X. , Yin P. , Bao X. , Tang H. , and Kang X. , Profile of Remimazolam in Anesthesiology: A Narrative Review of Clinical Research Progress, Drug Design, Development and Therapy. (2022) 16, 3431–3444, 10.2147/dddt.s375957. 36213379 PMC9541296

[13] Jiang W. , Cao J. , Xue Z. Y. , and Qian B. , Clinical Effects of Remimazolam Tosilate Combined With Remifentanil in Hysteroscopic Surgery, Jiangsu Medicine. (2023) 49, no. 7, 677–680.

[14] Kim K. M. , Remimazolam: Pharmacological Characteristics and Clinical Applications in Anesthesiology, Anesthesiology and Pain Medicine. (2022) 17, no. 1, 1–11, 10.17085/apm.21115. PMC8841266 35139608

[15] Li X. L. , Li W. X. , Huang J. B. , and Feng C. Q. , Observation of Anesthetic Effectiveness of Remimazolam Combined With Fentanyl for Hysteroscopy Examination, Harbin Medicine. (2023) 43, no. 3, 46–49.

[16] Li X. L. , Li W. X. , Zhang Q. L. , and Feng C. Q. , Application Effect of Remazolam Combined With Sufentanil Laryngeal Mask General Anesthesia in Hysteroscopy and Its Influence on Resuscitation Quality of Patients, Clinical Rational Drug Use. (2023) 16, no. 20, 108–110.

[17] Li C. K. , Anesthesia Efficacy and Safety Analysis of Remimazolam Tosilate in Pain-Free Hysteroscopy Examination, Chinese Journal of Drug Abuse Control. (2022) 28, no. 4, 458–462.

[18] Peng S. , Application of Remazolam in Painless Hysteroscopic Surgery and Analysis of Patient Status Index, 2022, Central South University.

[19] Qing C. H. , Peng S. Q. , and Xu Q. W. , Effect of Remazolam Toluene Sulfonate Combined With Hydromorphone in Hysteroscopic Surgery, Clinical Medical Engineering. (2022) 29, no. 5, 619–620.

[20] Li J. Q. , Yang M. , and Zhang Q. , The Influence of Different Doses of Remimazolam on the Requirement of Propofol During Hysteroscopy, Journal of Clinical and Experimental Medicine. (2024) 23, no. 17, 1902–1905.

[21] Wang Y. F. , Hou H. J. , and Hu N. G. , Effect of Remazolam Combined With Remifentanil in General Anesthesia for Hysteroscopic Surgery, Clinical Medicine. (2023) 43, no. 7, 43–46.

[22] Wu N. , Wang Z. M. , Chu H. J. , and Wang D. L. , Effects of Remazolam Besylate on Quality of Anesthesia Recovery and Cognitive Function in Patients Undergoing Hysteroscopic Surgery, Journal of Precision Medicine. (2022) 37, no. 1, 75–79+84.

[23] Zheng M. , Jia G. H. , and Liu J. , Effects of Remazolam Benzoate and Propofol on Anesthesia and Recovery Time in Hysteroscopic Surgery, Chinese Journal of Family Planning. (2022) 30, no. 1, 63–66.

[24] Noor N. , Legendre R. , Cloutet A. , Chitneni A. , Varrassi G. , and Kaye D. , A Comprehensive Review of Remimazolam for Sedation, Health Psychology Research. (2021) 9, no. 1, 10.52965/001c.24514. PMC8567764 34746482

[25] Zhang F. , Chang H. , Qing W. , Yu R. , Liao Q. , and Tong J. , Remimazolam Tosylate Combined With Low-Dose Propofol Improves Sedation and Safety in Hysteroscopy, Drug Design, Development and Therapy. (2022) 16, 4101–4108, 10.2147/DDDT.S390403. 36471692 PMC9719264

[26] Zhang S. , Wang J. , Ran R. , Peng Y. , and Xiao Y. , Efficacy and Safety of Remimazolam Tosylate in Hysteroscopy: A Randomized, Single-Blind, Parallel Controlled Trial, Journal of Clinical Pharmacy and Therapeutics. (2022) 47, no. 1, 55–60, 10.1111/jcpt.13525. 34655087

[27] Zhang X. , Li S. , and Liu J. , Efficacy and Safety of Remimazolam Besylate Versus Propofol During Hysteroscopy: Single-Centre Randomized Controlled Trial, BMC Anesthesiology. (2021) 21, no. 1, 10.1186/s12871-021-01373-y. PMC8135983 34016045

[28] Sneyd J. R. , Gambus P. L. , and Rigby-Jones A. E. , Current Status of Perioperative Hypnotics, Role of Benzodiazepines, and the Case for Remimazolam: A Narrative Review, British Journal of Anaesthesia. (2021) 127, no. 1, 41–55, 10.1016/j.bja.2021.03.028. 33965206

[29] Lee A. and Shirley M. , Remimazolam: A Review in Procedural Sedation, Drugs. (2021) 81, no. 10, 1193–1201, 10.1007/s40265-021-01544-8. 34196946

[30] Morimoto Y. , Efficacy and Safety Profile of Remimazolam for Sedation in Adults Undergoing Short Surgical Procedures, Therapeutics and Clinical Risk Management. (2022) 18, 95–100, 10.2147/tcrm.s304556. 35140469 PMC8819169

[31] Sneyd J. R. and Rigby-Jones A. E. , Remimazolam for Anaesthesia or Sedation, Current Opinion in Anaesthesiology. (2020) 33, no. 4, 506–511, 10.1097/aco.0000000000000877. 32530890

[32] Stehr-Pingel L. , Maagaard M. , Tvarnø C. D. , Andersen L. P. K. , Andersen J. H. , and Mathiesen O. , Remimazolam for Sedation: A Protocol for a Systematic Review With Meta-Analysis, Trial Sequential Analysis, and GRADE Approach, Acta Anaesthesiologica Scandinavica. (2023) 67, no. 10, 1432–1438, 10.1111/aas.14316. 37580880

